# What Happens When Donors Pull Out? Examining Differences in Motivation Between Health Workers Who Recently Had Performance-Based Financing (PBF) Withdrawn With Workers Who Never Received PBF in the Democratic Republic of Congo

**DOI:** 10.15171/ijhpm.2019.55

**Published:** 2019-07-13

**Authors:** Rishma Maini, Julia Lohmann, David R. Hotchkiss, Sandra Mounier-Jack, Josephine Borghi

**Affiliations:** ^1^Faculty of Public Health Policy, London School of Hygiene and Tropical Medicine, London, UK.; ^2^Faculty of Medicine, Heidelberg Institute of Global Health, Heidelberg University, Heidelberg, Germany.; ^3^School of Public Health and Tropical Medicine, Tulane University, New Orleans, LA, USA.

**Keywords:** Motivation, Health Workers, Performance-Based Financing, Democratic Republic of Congo, Factor Analysis

## Abstract

**Background:** A motivated workforce is necessary to ensure the delivery of high quality health services. In developing countries, performance-based financing (PBF) is often employed to increase motivation by providing financial incentives linked to performance. However, given PBF schemes are usually funded by donors, their long-term financing is not always assured, and the effects of withdrawing PBF on motivation are largely unknown. This cross-sectional study aimed to identify differences in motivation between workers who recently had donor-funded PBF withdrawn, with workers who had not received PBF.

**Methods:** Quantitative data were collected from 485 health workers in 5 provinces using a structured survey containing questions on motivation which were based on an established motivation framework. Confirmatory factor analysis was used to verify dimensions of motivation, and multiple regression to assess differences in motivation scores between workers who had previously received PBF and those who never had. Qualitative interviews were also carried out in Kasai Occidental province with 16 nurses who had previously or never received PBF.

**Results:** The results indicated that workers in facilities where PBF had been removed scored significantly lower on most dimensions of motivation compared to workers who had never received PBF. The removal of the PBF scheme was blamed for an exodus of staff due to the dramatic reduction in income, and negatively impacted on relationships between staff and the local community.

**Conclusion:** Donors and governments unable to sustain PBF or other donor-payments should have clear exit strategies and institute measures to mitigate any adverse effects on motivation following withdrawal.

## Background

Human resources for health are one of the core pillars of health systems,^1^ and the performance of health workers directly affects the quality of health services. Knowledge and competency are not the only influences of health worker performance.^2,3^ Studies have confirmed that there are differences in practice between what health workers “know” should be performed, and what they actually “do,” and this is termed the “know-do” gap.^4^ Alongside other factors including enabling working conditions, motivation is thought to be one bridge in overcoming this gap,^5^ and is often defined as the “degree of willingness of an individual to exert and maintain an effort towards attaining organisational goals.”^6^

In developing countries, health workers face many challenges to delivering services, including inadequate resources, supervision and training. In such settings, highly motivated workers will attempt to overcome such obstacles in order to be as productive as possible. Addressing poor health worker motivation can therefore lead to significant gains in efficiency and performance.^7-9^

One way of influencing motivation is through incentives, which may be financial or non-financial. Financial incentives are monetary rewards given to a worker,^10^ while non-financial incentives include: career development, resource availability, hospital management, supervisory support and recognition.^11-14^ With respect to financial incentives, performance-based financing (PBF) can be employed, and involves the transfer of funds (either totally or in part) to health workers based on their attaining a pre-defined level of performance. However, while PBF is expected to increase motivation and therefore effort, in some low-income countries and fragile states, the effects of introducing PBF upon motivation have produced mixed results; workers in Rwanda reported increased levels of motivation under a PBF scheme,^15,16^ while a study in Afghanistan indicated that PBF did not have a bearing on motivation and performance.^17^ In Malawi, PBF appeared to impact upon health worker motivation through different mechanisms, for example by improving their working environment.^18^ The authors recommended PBF schemes should be designed and implemented in anticipation of the different ways they will influence motivation, in order to ensure effects on motivation can be maximised.^18,19^

Donors often initially fund PBF in low-income country settings. According to the World Bank’s Results Based Financing for health website, the Health Results and Innovation Trust Fund has committed US$385.6 million to funding PBF programmes in 29 countries, which is linked to US$2 billion in financing from the International Development Association.^20^ Yet, with the exception of a few countries including Rwanda, Republic of Congo, and Burundi,^21-23^ the availability of government financing to take over such donor-funded schemes is not always assured. The volatility and unreliability of foreign financing could leave developing countries vulnerable if donors were to withdraw their aid.

Given financing is not always secure, knowledge of the implications of terminating such donor-funded payments is urgently needed to counteract any potentially harmful consequences for health worker motivation. To date, the authors are only aware of one study which was undertaken by Huillery and Seban,^16^ that examined the withdrawal of donor-funding in a low-income country which was the Democratic Republic of Congo (DRC). It compared health worker motivation between 2 groups – one receiving an exclusively performance-based payment, and the other group receiving a fixed payment of the same amount. The findings indicated that the motivation of workers was higher under the performance payment compared to the fixed payment, but following the removal of both payments, worker motivation was lower in the group which had received the performance payment. The study further found that the previous PBF group placed greater importance on financial motives than on non-material motives compared to the fixed payment group, which could not be attributed to a decrease in worker income.

Our study goes beyond the work undertaken by Huillery and Seban in that it examines differences observed across a vast range of dimensions of motivation between 2 groups of health workers; one group who recently experienced the removal of a donor-funded PBF scheme (comprising of both a fixed and variable payment related to performance) and another group of workers who were not exposed to PBF. The study also qualitatively explores how the withdrawal of PBF affected health workers, and reasons behind any differences identified between the 2 groups.

### Study Setting

Like many fragile states, the DRC struggles to provide basic healthcare to its citizens.^24^ Despite the domestic health budget mainly serving to finance health workers, few public sector health workers receive their government salary at all.^25,26^ Consequently, several donors have implemented PBF in an attempt to motivate the health workforce and enhance quality of care.^27^

Between 2008 and 2013, the Department for International Development (DFID) provided funding to 2 international non-governmental organisations (NGOs) to support health centres and hospitals to deliver a package of basic primary health services. The programme was called Access To Healthcare (ATH), and was implemented in 20 health zones in the provinces of Kasai Occidental, Province Orientale, Maniema, and South Kivu in the DRC^[1]^. The programme heavily subsidised user fees and additionally implemented PBF, involving monthly supplemental fixed payments to public sector health workers of $75 plus a monthly performance-based payment ($25). Facilities were scored against a series of quantitative performance targets, for example the attainment of 80% coverage for assisted births. Workers only received the performance payment if their facility achieved above a certain total score. Verification of performance was conducted by the implementing partners, who compared reported health service indicators with those found in health facility registers, and also visited a sample of patients recorded on the register to cross-check accuracy of information.

**Figure 1:**
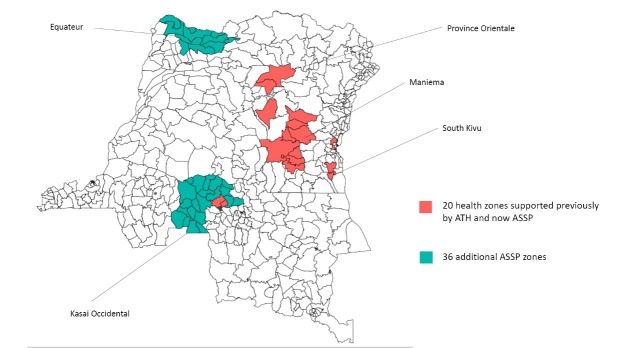
Health Zones in the DRC Supported by Different Health Programmes Funded by DFID.Abbreviations: ASSP, Access to Primary Healthcare (2013-2018); ATH, Access to Healthcare (2008-2013); DRC, Democratic Republic of Congo; DFID, Department for International Development.

At the end of ATH in March 2013, DFID commenced a follow-on programme called Accès Aux Soins de Santé Primaire (Access to Primary Health Care or ASSP) in a total of 56 health zones – the 20 health zones of ATH, and a further 11 zones in Equateur and 25 zones in Kasai Occidental (Figure 1).

The ASSP programme continues to support the provision of essential primary health services. However, during the first year of ASSP, PBF (both the supplemental fixed payment and performance payment) to health workers was phased out in the 20 health zones of the previous ATH programme by $25 every 3 months. Firstly, the $25 performance-based component of the payment was withdrawn 3 months after the start of the new ASSP programme (in July 2013). Then the fixed income component was reduced by $25 every 3 months thereafter, so all donor-funded payments to workers had ended by March 2014. Therefore, after 5 years of implementation under ATH, PBF was removed in a structured and gradual way under the successor programme ASSP. This occurred in tandem with a marginal increase in the user fee tariff, in an effort to help substitute health workers’ loss of revenue.

### Conceptual Framework

Over 70 years of research particularly in psychology and behavioural economics have led to numerous definitions, theories, and taxonomies of motivation in general and work motivation in specific.^28^ Motivation is usually viewed as a complex, multi-dimensional construct. For instance, motivation is often distinguished into different forms by its drivers (eg, extrinsic versus intrinsic motivation^28,29^; Hackman and Oldham’s Job Characteristics Model)^30^ or by its locus of causality (Self-Determination Theory).^31^ Other theories such as goal setting theory are concerned with individuals’ cognitive processes leading to motivated behaviour, it’s direction, intensity, and duration.^28^ Along with this variety of conceptualisations, numerous motivation measurement tools have been developed and validated, attempting to capture motivation either by asking directly about, or by asking about or observing proxies (eg, asking for factors assumed to be closely associated with motivation, such as working conditions; observing behaviours assumed to be the consequence of motivation).^32,33^

**Figure 2:**
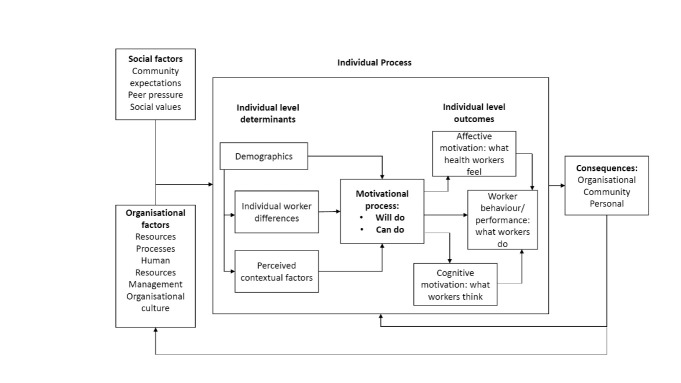
Franco’s Conceptual Framework of Motivational Determinants, Outcomes and Consequences. Source Franco et al.^40^

In low- and middle-income country (LMIC) health systems, interest in understanding the work motivation of the healthcare workforce is recent, but more and more studies are being conducted and published.^18,19,34-39^ Motivation researchers in LMICs struggle not only with the many available conceptualisations and measures, but also with the fact that very few have been validated and/or customised to the specific cultural contexts and work settings.^33^ Notable exceptions include a Self-Determination Theory-based psychometric scale,^36^ as well as the Franco framework.^8,40,41^ The Franco framework, which was also used in this study, is to date the most widely used framework in the current body of literature on work motivation of health workers in LMICs and captures motivation indirectly through various assumed motivational determinants and consequences at the individual, organisational and societal level (Figure 2). These determinants are described as either affecting the “will-do” component of motivation, the alignment of individual’s goals to that of the organisation, or the “can-do” component of motivation, which refers to the ability of the individual to mobilise resources to execute a task. Individual motivation outcomes are the result of the interaction between the “can-do” and “will-do” components of motivation, and can be affective, cognitive and behavioural. Affective outcomes concern health workers’ satisfaction, cognitive outcomes relate to health workers’ perceptions of their job, and behavioural outcomes relate to the performance of health workers. This study conceptualises motivation according to the Franco framework, measuring the impact of PBF withdrawal on various motivational determinants and consequences. In the following sections, we use the term ‘dimensions’ to refer to the individual-level determinants and consequences through which motivation is defined and measured in the Franco framework.

## Methods

The study employed a mixed methods triangulation design. In line with Creswell and colleagues,^42,43^ we use the term triangulation to refer to the use of different methods of data collection and analysis to explore different aspects of the ‘phenomenon.’ Specifically, we use quantitative methods to quantify motivational factors, and qualitative methods to elucidate the reasons why there may have been differences in motivation scores between health workers who had previously been exposed to PBF and health workers who had not been exposed. Quantitative data were collected through a health worker survey and allowed a comparison of scores on motivation dimensions between workers who had experienced PBF withdrawal with workers who had never received PBF; in other words, those who had been working in ATH areas prior to ASSP (previous PBF group) with workers who had not been covered by the ATH programme (non-PBF group). This latter group included workers covered by the ASSP programme as well as those working in non-ASSP health zones to enable a larger sample for comparison, and implementation of ASSP was still at an early stage. Qualitative data were collected using semi-structured interviews. Triangulation of quantitative and qualitative findings occurred at the interpretative stage.

### Quantitative Data

Using Franco’s framework,^6^ dimensions of motivation were identified. To identify questionnaire items to measure each dimension, an extensive review of health worker motivation surveys was performed and appropriate items collected.^11,39,41,44-53^ Identified items and dimensions were discussed with development partners to confirm the selection was relevant to the setting. However, it was not possible to cover all specified dimensions of motivation according to the Franco framework given other competing priorities of the health worker survey. As a result, we had to be conservative on the number of questions and therefore dimensions of motivation that we could measure. The final dimensions selected were those deemed to be of greatest importance and applicability to the DRC context.

The final questions were then incorporated into the health worker survey, which also gathered demographic information on health workers, including: age, gender, cadre, educational attainment, number of years worked, and number of financial dependents. Small revisions were made following a pre-test of the survey in 2 non-study facilities in Kinshasa and a facility in Bas-Congo. All items were answered on a 5-point Likert scale, with certain question responses worded “strongly disagree” to “strongly agree” and others worded “very dissatisfied” to “very satisfied.” The response “not applicable” was included for items where it was possible the question was not relevant.

### Sampling

**Figure 3:**
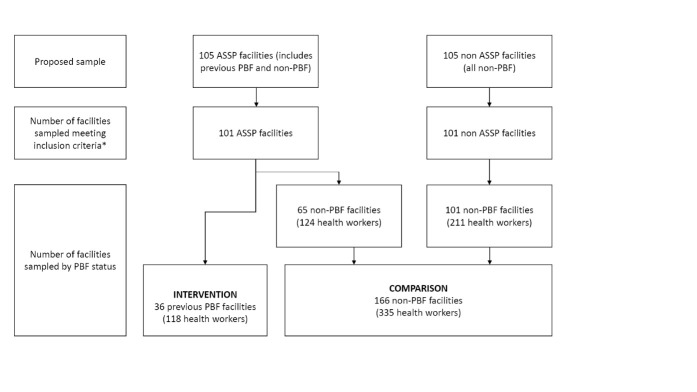
Diagram Showing Sampling of Facilities Under the Baseline Evaluation and the Selection of Intervention and Comparison Groups for This Study. Abbreviations: ASSP, Access to Primary Healthcare (2013-2018); ATH, Access to Healthcare (2008-2013); PBF, Performance-based financing.
* Some surveyed facilities dropped as health workers did not meet inclusion criteria.

The surveys were undertaken as part of a baseline evaluation of ASSP.^54^ Province Orientale and Maniema were combined to form one sampling domain, Kasai Occidental and Kasai Oriental formed another, and Equateur was its own sampling domain. 105 primary care facilities in ASSP areas were randomly sampled and matched with 105 facilities on urban/rural status and catchment population size in areas where ASSP was not operational (35 intervention and 35 control facilities in each sampling domain). Therefore, although this study was nested within the ASSP baseline evaluation, the sampling of facilities and workers had not taken into account coverage of the previous ATH programme ie, whether workers belonged to the “previous PBF group” or “non-PBF group.” This latter group included workers working in non-ASSP health zones as well as ASSP zones which had not been covered by ATH. As a result, there was an imbalance in the final sample of former PBF workers compared to non-PBF workers, with a far greater number of the latter. For this study, the intervention group was workers in ASSP that had received PBF, and the comparison group were workers (ASSP or non-ASSP) who had not received PBF. Figure 3 illustrates the results of facility sampling by PBF status.

All workers providing clinical services and on duty on the day of the survey were interviewed from sampled facilities. The head of the facility also completed a facility survey to determine the total number of staff, population served, and the number of services provided at the facility.

Data were collected between April and May 2014, which meant that any workers sampled in the previous PBF group had stopped receiving any donor-funded payments for at least one month. The Kinshasa School of Public Health in collaboration with Tulane University hired and trained data collectors from each of the sampled provinces to administer the surveys. Data collectors explained the purpose, confidentiality and anonymity of the study to each health worker in obtaining informed consent to participate in the survey.

### Statistical Analyses

Survey data were double-entered into the computer database CSPro for verification before being imported into and analysed in Stata 13 and R 3.4.2 statistical software. Descriptive statistics were used to explore the demographic characteristics of health workers and facility characteristics overall, and differences between the previous and non-PBF groups assessed using chi-square and *t* tests. For the motivation dimensions, psychometric item analysis, examining item distributions, summary statistics, and correlation patterns, was undertaken in Stata 13 for both the overall sample and the previous PBF and non-PBF subgroups (see Supplementary file 1, Table S1). Items which clearly did not correspond well with the other items pertaining to their intended dimension were dropped (see Supplementary file 2, Table S2). The final 11 motivation dimensions, along with hypotheses on how PBF withdrawal may affect these, are listed in Table 1.

**Table 1:** Final Motivation Dimensions, Link to Franco Framework, and Hypothesis on how PBF Removal May Affect Dimensions

**Link to Franco Framework**		**Dimension (Link to Franco Framework)**	**Description of Dimension**	**How Expected Withdrawal of the Donor-Funded Payment May Affect Dimension**	**Rationale**	**No. of Items**
Determinants of motivation	Individual-level	Conscientious-ness (affective motivation)	Perception of level of discipline, effort and care put into work	Decrease	The introduction of PBF has been shown to increase worker effort^55^, as well as reduce absenteeism.^56^ The study by Huillery and Seban showed that staff attendance reduced significantly following the withdrawal of PBF.^16^ It also reduced in the group where the fixed payment was removed but not to the same extent. We therefore hypothesise that withdrawal of donor funding will lead to reduced conscientiousness.	9
		Pride (affective motivation)	Pride associated with working at the facility	Decrease	According to incentive theory, the introduction of financial rewards may ”crowd out” intrinsic motivation (which includes feelings of pride); this crowding out phenomenon is more likely when employees have high initial levels of intrinsic motivation, eg, when pride in one's work is high and the activity is interesting.^57^ The evidence around this is contradictory with respect to PBF.^58^ For example, health workers in Bubanza province of Burundi claimed that PBF helped to generate pride and professionalism, while in another Burundian province, the PBF bonus was gradually perceived as a right and a fixed extra which may have led to less intrinsic motivation.^59^ In addition, the theory does not predict what happens to intrinsic motivation when the reward is withdrawn, If there is a shift in fundamental motivation composition caused by financial rewards, one may expect pride would remain low rather than increase on withdrawal of the reward.	4
		Extrinsic motivation (cognitive motivation)	Importance health workers place on external rewards	Increase	The importance placed on external rewards by workers receiving PBF has been observed in other studies, and manifest through workers prioritising tasks linked to higher incentives,^15,60,61^ or gaming.^15,62^ Huillery and Seban also found that more attention was paid to financial motives relative to intrinsic motives following the withdrawal of PBF, and that this was not due to the decrease in worker salary.^16^ Therefore, it is expected that extrinsic motivation would increase.	1
		Sufficiency of income (affective motivation)	Degree to which health workers feel like their income is sufficient given their basic monetary needs	Decrease	Although there is no evidence on this from previous studies, it is expected that workers will perceive the sufficiency of income to be less following withdrawal of PBF, as workers would be expected to perform the same tasks for less money overall.	1
		Income reflects effort (affective motivation)	Degree to which health workers feel income received reflects the amount of effort put into work	Decrease	Although there is no evidence on this from previous studies, it is expected that workers will likely perceive their (reduced) income as less appropriate in relation to effort following withdrawal of PBF, as workers would be expected to perform the same tasks for less compensation.	3
	Organisational level	Training (processes)	Satisfaction with training received and opportunities for training	No effect	The PBF scheme did not include training and so its withdrawal would not be expected to have an effect on workers’ satisfaction with training. Levels of training offered would be the same in the previous PBF and non-PBF groups.	3
		Tasks (processes)	Satisfaction with workload and variety of tasks performed	No effect	The withdrawal of the PBF scheme was not accompanied by a change in service organisation or workload. Therefore, no difference in this dimension was expected between the previous PBF and non-PBF groups.	4
		Availability of equipment/ Supplies (resources)	Satisfaction with availability of resources such as equipment, medical supplies and medications	No effect	The PBF scheme did not include increased equipment or supplies and so its withdrawal would not be expected to have an effect on workers’ satisfaction with the availability of equipment or supplies. The availability of equipment and supplies was not be expected to vary between the non-PBF and PBF groups.	3
		Organisational culture (organisational culture and human resources management^a^)	Satisfaction with relationships with colleagues and management of the facility	Decrease	PBF has been shown to increase levels of collegial support in Mozambique.^63^ The potential volatility of PBF was noted to be a source of stress for the heads of facilities in the DRC,^16^ so the withdrawal was anticipated to negatively affect health workers’ satisfaction with interpersonal relationships at work. PBF has recently been shown by some studies to improve supervision due to the levels of reporting and monitoring required.^63,64^ Therefore, it was plausible that this could reverse with the removal of PBF, potentially leading to lower levels of supervision.	4
	Community level	Community relationships (consequence at community level)	Satisfaction with relationships with local leaders in the community	Decrease	In Rwanda, PBF was evaluated to have a positive effect on patient satisfaction,^65^ which could improve community relationships with health providers. Yet, the presence of PBF did not have any impact on patient satisfaction according to Huillery and Seban’s study.^16^ Therefore, withdrawal either expected to have no effect, or result in lower satisfaction from the community.	1
Outcomes	Behaviour	Turnover intention (cognitive motivation)	Intention to leave the facility	Increase	Lack of satisfaction with salary (amongst other job aspects such as career opportunities) has been associated with higher turnover intention in a number of studies.^66-68^ Therefore, a reduction in income through PBF withdrawal would be likely to increase staff turnover intention.	1

Abbreviations: PBF, performance-based financing; DRC, Democratic Republic of Congo. ^a^Although the dimensions “Management” and “Organisational Culture” are treated as separate entities within the conceptual framework, they were merged together as items relating to management were worded in a way that they reflected organisational culture.

Prior to the analysis, in the few cases where individuals missed a response to an item, missing responses were replaced by imputation of the median value of their responses to other items pertaining to the same dimension. Confirmatory factor analysis, which was carried out in R 3.4.2 using a robust maximum likelihood estimator, indicated a good model fit for the 11-factor model (*χ* ^2^(476) = 816, *P*  = .000; RMSEA = 0.040, *P* (RMSEA<.05) = 1.000; CFI = 0.902; SRMR = 0.045), confirming that the 34 motivation-related items measured the 11 motivation dimensions as intended. Cronbach alpha was greater than 0.60 for all dimensions with 3 or more items. Factors with more than one item were also tested for measurement invariance across both PBF groups. Measurement invariance testing aims to confirm that the scale has the same measurement properties in different subsamples and scores can therefore meaningfully be compared across samples. Establishing measurement invariance involves a hierarchy of testing, which include tests of weak and strong invariance.^69^ Strong invariance was identified for the dimensions: ‘conscientiousness,’ ‘pride’ and ‘training,’ weak invariance for ‘organisational culture’ and ‘tasks,’ while only ‘availability of equipment/supplies’ and ‘income reflects effort’ were not invariant and differences between subsamples therefore need to be interpreted with caution.

Scores for each dimension were then calculated as unweighted means of responses to items within each dimension, as within each dimension, item-factor loadings were of approximately the same magnitude. Multiple linear regression models with an ordinary least squares estimator as the standard were used to test for significant differences in motivation scores between workers from the non-PBF group and workers in the previous PBF group, controlling for health worker and facility characteristics including: age, gender, health worker cadre, education, years worked in current position, location, type of facility, number of services provided, and presence of the ASSP programme. Table S3 in Supplementary file 3 indicates how different characteristics may have influenced motivation scores, based on the global evidence.

The dependent variable was the score on each dimension. Standard errors (SEs) were clustered at facility-level and ordinary least squares assumptions checked using regression diagnostics.

### Qualitative Data

Qualitative data were collected in November 2014 in Kasai Occidental. Two health zones where workers had previously received PBF under the ATH programme were selected purposively, as well as another 2 health zones where workers had never received PBF. Two facilities in each health zone most easily accessible by road were then chosen by the researchers. One female and 1 male nurse were purposively selected from a single facility in each of the 4 health zones, as nurses are the main cadre present in primary care facilities.^70^ They were then interviewed subject to consent and meeting a further inclusion criterion, which was to have been based at the same facility for at least 1 year. Workers identified for interview in the non-PBF group were excluded if they had previously received PBF over the past 5 years. In-depth interviews were conducted using a semi-structured interview guide. As the purpose was to help further understand quantitative findings, perceptions and differences in the pre-identified dimensions of motivation were explored. The guide was pre-tested in a health centre before being finalised. The primary author (RM) and an experienced local qualitative researcher familiar with the cultural context performed all 16 interviews in French, and these were audio-recorded subject to participant consent. None of the respondents declined to participate. Interviews lasted one to 2 hours and were undertaken in a private room within facilities to maintain confidentiality.

### Analysis

Audio recordings were transcribed in French by the local researcher to maximize accuracy and reviewed by RM, thus enabling both researchers to familiarise themselves with the data prior to coding. Nvivo 10 software was used to manage the qualitative data. Thematic analysis^71,72^ was employed using both deductive coding based on the dimensions measured in the quantitative analysis as codes, as well as inductive coding allowing the framework to evolve as interviews were analysed. RM and the local researcher independently initially coded the transcripts and then met for an analysis session which involved discussing the themes generated. During the analysis session, it was clear that the researchers’ individual interpretations of the transcripts were very similar; any differences that did occur were resolved by discussion. Results were than compared and contrasted for the previous PBF and non-PBF groups. In writing up the findings, RM translated quotes from French into English.

## Results

### Quantitative Analysis

A total of 485 workers were interviewed and no one declined to participate in the survey. Three facilities did not meet the inclusion criteria as they were private facilities, and 23 respondents were not classified as health workers. On elimination, this left 453 respondents from 202 facilities for analysis.

### Descriptive Statistics

The results of this study compare the PBF group with the non-PBF group; although the latter group comprised both ASSP and non-ASSP facilities, on testing for differences in characteristics, the only significant differences identified were at facility-level for provincial location and the number of services provided, as shown in Table 2. However, there were no differences between health worker characteristics. Given the ASSP programme had not fully commenced implementation when sampling occurred, it is not surprising that characteristics were broadly similar. Hence they were merged into one comparison group for analysis. Table 2 presents the characteristics of sampled facilities, 36 of which were in areas where PBF had been operational. There were significant differences in the numbers of previous PBF and non-PBF facilities between provinces, as most of the previous PBF facilities were based in Maniema and the non-PBF facilities in Equateur. The majority of facilities were located in rural areas, with previous PBF facilities having a significantly higher number of personnel and offering more services compared to non-PBF facilities.

**Table 2:** Characteristics of Previous PBF and Non-PBF Facilities Sampled

**Facility characteristics**	**Total (n = 202)**	**Non-PBF (n = 166)**	**Previous PBF (n = 36)**	**Test Statistic,** ***P*** **Value**
Number of facilities	202	166	36	
Facility location				
Urban	14.8%	12.0%	25.0%	χ^2^ (1) = 5.10, *P* = .024
Rural	85.2%	88.0%	75.0%	
Province				
Equateur	33.7%	41.0% (ASSP: 52.3%, non-ASSP: 33.7%)	0.0%	χ^2^ (4) = 100.30, *P* < .0001 (Between non-PBF facilities: χ^2^ (4) = 39.22, *P* < .0001)
Kasai Occidental	28.2%	32.5% (ASSP: 47.7%, non-ASSP: 22.8%)	8.3%	
Kasai Oriental	6.0%	7.2% (ASSP: 0%, non-ASSP: 11.9%)	0.0%	
Maniema	17.3%	5.4% (ASSP: 0%, non-ASSP: 8.9%)	72.2%	
Province Orientale	14.9%	13.9% (ASSP: 0%, non-ASSP: 22.8%)	19.4%	
Type of facility				
Health centre/health post	85.2%	85.5%	87.0%	χ^2^ (1) = 0.28, *P* = .598
Reference health centre	14.4%	14.5%	13.9%	
	**Mean**	**Mean, SE**	**Mean, SE**	
Number of different services provided by facility (eg, antenatal care, vaccinations etc) (Total n = 194, non-PBF = 160, PBF = 34)	6.97^a^	6.83 ± 0.13^a^ ASSP: 6.35 ± 1.50; non-ASSP: 7.16 ± 1.62	7.65 ± 0.22^a^	t = -2.76, *P* = .0064 (Between non-PBF facilities: t = -3.22, *P* = .0015)
Total clinical staff present on the day	6.31	5.45 ± 0.32	10.28 ± 0.83	t = -6.09, *P* < .0001
Population catchment for area (Total n = 163, non-PBF = 135, PBF = 28)	2710.31^a^	2725.64 ± 236.40^a^	2636.39 ± 431.04^a^	t = 0.16, *P* = .8725

Abbreviations: PBF, performance-based financing; SE, standard error; ASSP, Access to Primary Health Care. ^a^N less than total number of facilities for some variables due to missing values.

Most health workers surveyed were nurses and male (Table 3). The majority had attained either a primary or secondary level of school education and mean job tenure was almost 9 years. There were no significant differences in the composition of cadres working in previous PBF facilities compared to the non-PBF group, although previous PBF workers were significantly older and more likely to have had a university/post-secondary school education.

**Table 3:** Demographic Characteristics of Previous PBF and Non-PBF Health Workers

**Characteristics**	**Total (n = 453)**	**Non-PBF (n = 335)**	**Previous PBF (n = 118)**	**Test Statistic,** ***P*** **Value**
Gender				
Male	69.3%	70.2%	67.0%	χ^2^ (1) = 0.14, *P* = .712
Female	31.7%	29.8%	33.0%	
Education				
Primary/secondary school	60.7%	65.1%	48.3%	χ^2^ (2) = 11.52, *P* = .003
University/post-secondary school	33.1%	29.6%	43.2%	
Not specified	6.2%	5.4%	8.5%	
Cadre				
Doctor	0.9%	0.6%	1.7%	χ^2^ (2) = 1.20, *P* = .549
Nurse	89.8%	90.2%	89.0%	
Other clinical workers	9.3%	9.2%	9.3%	
	**Mean**	**Mean, SE**	**N, Mean, SE**	
Age	40.01	39.41 ± 0.53	41.64 ± 0.87	t = -2.19, *P* = .029
Number of financial dependents (Total n = 437, non-PBF = 320, PBF = 117)	8.87^a^	8.69 ± 0.26^a^	9.33 ± 0.38^a^	t = -1.29, *P* = .1964
Years worked in current position (Total n = 444, non-PBF = 327, PBF = 117)	8.93^a^	8.68 ± 0.46^a^	9.60 ± 0.87^a^	t = 1.05, *P* = .2956

Abbreviations: PBF, performance-based financing; SE, standard error. ^a^N less than total number of facilities for some variables due to missing values.

### Comparison of Motivation Scores Between Previous PBF and Non-PBF Groups

Mean and median composite scores for the dimensions of motivation overall and by PBF status are shown in Table 4. The dimension ‘satisfaction with sufficiency of income’ had the lowest mean score, whereas the highest mean scores were observed for items related to the dimension ‘level of conscientiousness.’ The largest difference in means between PBF groups was for ‘satisfaction with availability of equipment/supplies’ where the non-PBF group scored much higher. Mean and median scores for individual scale items are provided in the supplementary information (Table 1).

**Table 4:** Mean and Median Composite Scores for Dimensions of Motivation According to PBF Status

**Dimension**	**Overall**	**Non-PBF**	**Previous PBF**
	**Mean, SD (Median)**	**Mean, SD (Median)**	**Mean, SD (Median)**
Level of conscientiousness	4.10, 0.32 (4.00)	4.13, 0.32 (4.00)	4.04, 0.30 (4.00)
Level of pride	4.02, 0.50 (4.00)	4.07, 0.49 (4.00)	3.87, 0.52 (4.00)
Satisfaction with training	3.50, 0.81 (3.67)	3.59, 0.78 (4.00)	3.26, 0.85 (3.33)
Satisfaction with tasks	3.61, 0.62 (3.75)	3.56, 0.64 (3.75)	3.74, 0.53 (4.00)
Satisfaction with availability of equipment/supplies	2.29, 0.93 (2.00)	2.47, 0.91 (2.33)	1.76, 0.78 (1.67)
Satisfaction with sufficiency of income	1.71, 0.65 (2.00)	1.79, 0.67 (2.00)	1.47, 0.55 (1.00)
Satisfied that income reflects effort	2.03, 0.74 (2.00)	2.17, 0.76 (2.00)	1.64, 0.49 (1.67)
Satisfaction with organisational culture	3.83, 0.55 (4.00)	3.87, 0.55 (4.00)	3.72, 0.55 (4.00)
Satisfaction with community relationships	4.00, 0.72 (4.00)	4.04, 0.74 (4.00)	3.91, 0.64 (4.00)
Level of turnover intention	3.00, 1.25 (4.00)	3.07, 1.21 (4.00)	2.82, 1.34 (3.00)
Level of extrinsic motivation	3.62, 1.07 (4.00)	3.50, 1.07 (4.00)	3.95, 0.99 (4.00)

Abbreviations: PBF, performance-based financing; SD, standard deviation. Dimensions scored on scale from 1-5. A high mean or median score indicates a higher level for that dimension eg, higher pride.

Testing for mean differences using regression and controlling for health worker and facility characteristics as described in the methods, we found that health workers in previous PBF facilities scored significantly lower on all dimensions except ‘satisfaction with tasks’ (no significant differences) and ‘level of extrinsic motivation’ (marginally significantly higher scores) compared to those in non-PBF facilities. Table 5 summarises the regression results (separate models were run for each dimension).

**Table 5:** Summary of Ordinary Least Squares Regression Results Examining Associations Between PBF Removal and Scores on Motivation Dimensions

**Factor**	**β**	***P*** **Value**	**95% CI**	**Constant**	**Pseudo R** ^2 ^
Level of conscientiousness	-0.20	<0.001^c^	-0.31 to -0.10	3.76	0.12
Level of pride	-0.43	<0.001^c^	-0.61 to -0.25	3.67	0.10
Satisfaction with training	-0.47	0.004^c^	-0.78 to -0.15	3.74	0.11
Satisfaction with tasks	-0.09	0.40	-0.29 to 0.12	3.33	0.07
Satisfaction with availability of equipment/supplies	-0.62	<0.001^c^	-0.95 to -0.29	2.51	0.19
Satisfaction with sufficiency of income	-0.24	<0.047^b^	-0.48 to -0.00	2.15	0.10
Satisfied that income reflects effort	-0.51	<0.001^c^	-0.78 to -0.23	3.04	0.17
Satisfaction with organisational culture	-0.23	0.020^b^	-0.43 to -0.04	3.74	0.11
Satisfaction with community relationships	-0.44	<0.001^c^	-0.64 to -0.25	4.03	0.07
Level of turnover intention	-0.49	0.045^b^	-0.96 to 0.01	3.14	0.09
Level of extrinsic motivation	0.40	0.058^a^	-0.01 to 0.80	3.19	0.07

Abbreviation: PBF, performance-based financing. ^a^*P*  ≤ .1, ^b^*P*  ≤ .05, ^c^*P*  ≤ .01.

### Qualitative Findings

Sixteen nurses in total were interviewed; 8 in each PBF group, of which 4 were male and 4 were female. Ages of respondents in each group were of similar ranges and those in previous PBF facilities had worked between 2 and 16 years at the facilities, while in the non-PBF group they had worked between one and 10 years. The sections that follow describe the process of PBF withdrawal and how it may have affected the individual, organisation and community, with due comparison to the non-PBF group where relevant.

### Process of Performance-Based Financing Withdrawal

The key complaint from the previous PBF group around the process of withdrawing the PBF scheme was that it had been both abrupt and poorly communicated.

*“When the new partner removed the prime (PBF scheme) we did not even know that they had removed the prime…we were not informed”* [Respondent 1: Male 42 years, previous PBF].

This led to some resentment from workers in the previous PBF group towards the NGO implementing the ASSP programme. Workers also felt it was the responsibility of international partners to finance them, rather than that of the government.

*“For me, I would like it to be as before…I wish, there was another partner (NGO/donor) that can take care of us, so we can receive money at the end of each month”* [Respondent 2: Female, 60 years, previous PBF].

Since the removal of the PBF scheme also coincided with a deliberate increase in the user fee tariff, this only served to magnify problems for health facilities. Nurses complained that the community had become used to the previous lower user fee tariff and so were less willing as well as less able to pay the new tariff.

*“Because people are already used to the free tariff…for them it’s a huge problem, even for the maternity here it was free, now it’s 1,500FC (1 USD), but for people to pay that, it’s becoming quite a problem”* [Respondent 3: Female, 37 years, previous PBF].

### Individual Health Worker Effects

### “Extrinsic Motivation,” “Income Reflects Effort,” and “Conscientiousness”

When asked why nurses were motivated to work in their profession, few differences in responses were detected between the previous PBF and the non-PBF groups. Financial incentives appeared to be an important driver of motivation for a substantial share of the health workforce, irrespective of PBF. Against this general importance of financial incentives, respondents from the previous PBF group perceived that when they were receiving donor-funded payments, staff attendance at the facility was high, nurses worked hard and patients were treated in a timely way.

*“When we were paid, it stimulated us to work a lot and work well, we worked a lot, as we were paid, we had to be able to reach the percentage that was asked… we followed the (performance) indicators”* [Respondent 4: Female 27 years, previous PBF].

Since the withdrawal of donor payments, workers confessed to putting less effort into their work. They reported high levels of staff absenteeism and admitted that they were less punctual in attending the facility, as they felt they were not receiving enough money.

Respondent: *“Before (when receiving PBF), I would work even if I had not eaten…Now (after PBF), I don’t work a lot. There are even health centres which do 10% out of 100%, they work only 10%....* ”

Interviewer: *“Why do they not work a lot?”*

*Respondent: “Because of money”* [Respondent 4: Female, 27 years, previous PBF].

In particular, nurses felt the amount of effort required by the job was no longer sufficiently rewarded.

*“With the work one does, it’s a tough job, you can be standing up for a long time during 2 or 3 hours and at the end of the month, what you receive, it’s not enough…”* [Respondent 5: Female, 48 years, previous PBF].

Stories of corruption were frequent in both groups. Many workers shared tales of other workers stealing medications, equipment and medical supplies, which they then sold privately, and recounted examples of where patients had been overcharged for services. Irrespective of PBF group status, workers did not always see this as wrong, and some considered it justifiable in certain cases. There seemed to be no effect of PBF withdrawal in this regard.

*“He is right because, for example, he has been working for 40 years at the centre…since he started working he has not even been recognised…by anyone or government…he is not paid … he has 8 children. …with the little he has…it is insufficient. He must pay for his rent, for hischildrens’ school uniforms, to feed his children, why when in this manner he could take money (illegally) from the facility”* [Respondent 6: Male, 35 years, non-PBF].

### “Sufficiency of Income”

The amount previously received under the PBF scheme had allowed workers to pay for their children’s school fees, save money and buy enough food to feed their family well. Following the withdrawal of the PBF scheme, the social circumstances of nurses dramatically changed. Many nurses started to borrow money from their relatives in order to continue to meet their own and their family’s basic needs.

*“I managed, I sought help from my family to buy a few things to start selling in order to feed myself. With the money from my family, it is not my own money”* [Respondent 7: Female, 28 years, previous PBF].

Workers in the non-PBF group felt the amount they received was insufficient, particularly those who had been working for several years but were still not salaried by the state.

*“I have worked for almost 3 years in this health centre, and I have never received a salary…which is why I am not happy…I only receive the user fees at the end of the month which we share between us”* [Respondent 6: Male, 35 years, non-PBF].

*“We work and then and the end of the month, we receive almost nothing”* [Respondent 8: Female, 48 years, non-PBF].

Nonetheless, they did not describe any instances of borrowing from other relatives or other behaviours to supplement income as described by the previous PBF group.

### Organisation-Level Effects

### “Organisational Culture”

The majority of workers in the previous PBF group found that their main source of revenue had shifted from PBF payments to user fees following the withdrawal of PBF. This was problematic insofar as the management and allocation of user fees had become a source of conflict since the withdrawal of the PBF scheme, despite what nurses described as generally good working relationships with their superiors.

*“We receive always the user fee because for example, you have 2 patients, you are 10 workers, you have to calculate the percentage, you will have how much? One can say you have 5000 francs (4.5 USD)…How are you going to share that?”* [Respondent 7: Female, 28 years, previous PBF].

Workers in non-PBF facilities where user fees had always been the main source of revenue, in contrast, seemed satisfied with the allocation of user fees.

*“Management at the centre is good...there is transparency…and the user fees are well managed”* [Respondent 6: Male 35 years, non-PBF].

### “Turnover Intention”

Although the PBF scheme had initially attracted workers to facilities, shortly after it ceased, there was a mass exodus of workers. Some left clinical work completely to work in commercial activities.

Respondent: *“There was even a mutiny of other nurses who left the health zone”*

Interviewer: *“You know the number of nurses who left?”*

Respondent: *“Yes”*

Interviewer: *“How many?”*

Respondent: *“There were nearly 10 nurses in all of the health zone.”*

Interviewer: *“Why was there a mutiny?”*

Respondent: *“Because they were not receiving the prime (performance payment), they were going to stay to do what?”* [Respondent 9: Male, 30 years, previous PBF].

Workers in the previous PBF group who stayed on in facilities stated this was either because: they had no other options of work available to them; they were waiting for another donor or NGO to start paying them; or they felt a strong commitment to their vocation and enjoyed working in their profession.

*“I am here because I love to treat people. If it wasn’t for my desire to work as a nurse, I would return to Katanga province, where life is better compared to here in Kasai”* [Respondent 9: Male, 30 years, previous PBF].

### Community-Level Effects

### “Community Relationships”

As a result of the re-increase in user tariffs following the withdrawal of the PBF scheme, workers perceived that many patients were not attending the previous PBF facilities but were instead seeking care elsewhere, often from traditional healers or private facilities, or not at all.

*“Now to have money, to come to the centre, it’s always a problem, they take traditional medicines at home”* [Respondent 4: Female, 27 years, previous PBF].

It also became clear during interviews that the effects associated with terminating the PBF scheme were not only influencing staff behaviour and motivation but were being felt by the community as well. For example, some nurses remarked that colleagues had become less welcoming and were even rude to patients since donor payments to workers had ceased.

*“They say even in front of the patients there, the patient comes, they say ‘no, leave there, I can’t treat you as I’m not paid’”* [Respondent 9: Male 30 years, previous PBF].

## Discussion

Based on existing conceptual and empirical work, we developed a scale to measure dimensions of motivation among health workers in the DRC. We then used this scale to assess differences in levels of motivation across each dimension for workers who had recently had PBF withdrawn with workers who had never received PBF, triangulating the results with the findings from qualitative interviews in order to shed more light on how the termination of a PBF scheme had affected worker motivation.

The previous PBF group scored significantly lower on almost all motivation dimensions. Exceptions included ‘satisfaction with tasks’ where there was no significant difference between groups, and ‘level of extrinsic motivation’ where workers in the previous PBF group scored higher (marginally significant). Theoretically, a more significant effect with the latter dimension may have been expected; according to “crowding out” theory; the introduction of monetary incentives may alter the composition of worker motivation, with workers becoming more driven by external rewards and less by intrinsic motivation.^35,73^

Qualitative interviews indicated that financial compensation may have been an important dimension of motivation for both groups. The quantitative analysis showed that workers in the previous PBF group had significantly lower scores for the dimension ‘satisfaction with sufficiency of income.’ A likely reason behind the lower scores is that during PBF, workers were guaranteed a certain level of income each month (at least $75) but since the withdrawal of the PBF scheme, the amount received was less predictable as it was dependent on the amount of user fees collected; Fox et al found the monthly variation in user fees received by facilities in Katanga province of DRC to be considerable.^26^ A previous study by the authors examining the different income levels and sources of health workers using data from the same health worker and health facility surveys confirms that the income derived from user fees was on average much lower than the PBF payments.^70^ However, despite some differences in the various sources of income received, the total mean and median income received for both the previous PBF and non-PBF groups were still similar (see Supplementary file 4, Table S4).

Nonetheless, workers in the previous PBF group seemed unable to have the same lifestyle they had enjoyed before the withdrawal of PBF. Staff were unhappy with their lower level of compensation post PBF, leading to significantly lower scores on ‘satisfaction that income reflects effort’; their conscientiousness deteriorated as a result, with reports of high levels of staff absenteeism and poor attitudes towards patients. Such effects are potentially very destructive and undermine the effective functioning of the health system. The reasons for the significant differences between groups for the dimensions ‘satisfaction with training,’ ‘satisfaction with tasks,’ ‘level of pride,’ and ‘satisfaction with availability of equipment/supplies’ yielded by the quantitative analysis, however, could not be identified during qualitative interviews.

Although it could not be confirmed quantitatively, respondents described a reduction in the number of staff working in facilities following the cessation of incentives. Yet, contrary to interviews and the initial hypothesis, the previous PBF group scored lower on ‘level of turnover intention.’ The average tenure of workers in the previous PBF group was also not significantly different to that of the non-PBF group. It may be that those workers with a high turnover intention in the previous PBF group had already left facilities soon after PBF removal, so those interviewed were more committed to staying. Remaining staff had to rely on user fees received at the facility-level as their main source of income, the distribution of which was more often a source of dispute in previous PBF facilities compared to non-PBF facilities. This was perhaps because staff from non-PBF facilities had not experienced the same recent loss of income and so were well accustomed to the income received from user fees. In parallel, relationships between previous PBF workers and the local community became strained, as the implementing partners of ASSP had introduced higher user fees in order to help substitute performance payments. According to nurses in the previous PBF group, the community were less likely to access care from facilities as user fees had increased. Furthermore, in some workers, the resentment towards receiving a reduced income manifested itself in their attitudes towards patients. This breakdown in the interface between the community and health workers was consistent with the quantitative analysis where previous PBF workers had significantly lowers scores for ‘satisfaction with community relationships.’

The findings in this study do concur with another study in the DRC which found that the withdrawal of donor-funded payments did reduce the overall motivation of workers.^16^ However, in this study we were able to measure differences between a group having had PBF withdrawn with a “control” group across several different dimensions of motivation and employed in-depth qualitative investigation to yield a more in-depth understanding around the differences observed. There are few other reports in the wider literature which examine the withdrawal of donor-funded payments in relation to performance and motivation. A DFID-supported health programme in Liberia attempted to withdraw the payment of salary supplements to health workers. Following this, clinic staff began charging high fees for services and sold drugs to private clinics to enhance their income, necessitating the eventual reinstatement of financial incentives. Although anecdotes concerning unethical behaviour such as stealing were common in both groups in our study, it was likely to have been under-reported; given the small number of interviews conducted, we were unable to draw any strong conclusions as to whether this had changed in the previous PBF group. In the United States and United Kingdom, the cessation of performance payments did not appear to adversely affect performance or quality of care.^74,75^ Nevertheless, unlike this study, they were unable to indicate any differences observed with a control group. In addition, the contribution of performance payments compared to base salaries and other income sources is likely to be far lower in high-income countries compared with low-income countries, limiting the generalisability of the findings.

The programme’s decision to terminate the PBF scheme was made on the premise that the government is responsible for public sector personnel remuneration, and that PBF funded by donors is not a sustainable solution. However, in the absence of any structural improvement in government payments to workers, immediate service needs still have to be met. Important lessons from this experience include: more careful thinking around the implications of withdrawing donor-funding for workers and their social circumstances, and ensuring that there is an effective communication strategy with workers and communities on such programmatic changes to mitigate any adverse reactions. Alternative, acceptable measures should also be instituted which still make it attractive for workers to stay and provide high quality services at facilities. As indicated by previous studies, workers are not exclusively motivated by financial factors.^8^ Attention to non-financial dimensions such as the ‘satisfaction with the availability of equipment/supplies’ and ‘satisfaction with training’ which were rated low in this study could serve to enhance motivation. Country governments and their international health partners should also consider whether the short-term advantages of introducing additional health worker payments outweigh the potential adverse long-term consequences if the chances of it being sustained in the long-term for a given context are low. In designing any future PBF schemes, a realistic and well thought out exit strategy should be planned from the beginning to prevent long-term dependence, and the existing financial environment of workers should also be reviewed to ensure that payments are not distortionary in the context of other payments received. For example, one way of ensuring PBF is more structurally and financially sustainable, is to blend it with other existing payment mechanisms (such as salaries)^76^ as opposed to implementing it as a stand-alone, vertical programme, as was the case here. Another study in the DRC found that health workers appeared to value salaries more than PBF payments,^26^ and so efforts to ensure all legitimate workers are remunerated by the state should be prioritised.^77^ Furthermore, with respect to PBF, donors may consider what specific sources of funds would be used for a PBF scheme in the event their funding cannot be sustained.

A strength of this study is that it supplemented the quantitative data with qualitative insights in order to corroborate and explain findings. We developed a motivation measurement scale customised to workers in the DRC, which may be of use to future studies wishing to measure the effects of different interventions on motivation. In doing so, we chose a proxy-based approach to operationalising motivation, in line with many prior studies. We acknowledge, however, that such an approach relies on a number of assumptions about relationships between proxies and motivation itself about which we cannot be fully certain.

There were several limitations to the study, the main limitation being it was a cross-sectional study making it difficult to causally attribute the lower motivation levels observed in the previous PBF group to the cessation of the PBF scheme. It is possible that the findings demonstrate that the results of PBF are not sustained when funds are withdrawn, and that motivation levels in this group had returned to baseline levels (pre-PBF). Workers from previous PBF facilities also had some significantly different characteristics compared to non-PBF workers; in particular, many of the PBF and non-PBF workers were located in facilities in different provinces. Therefore, the differences in context could also explain some of the differences in motivation observed. This was unavoidable as coverage of the PBF model was determined by the geographical focus of the previous health programme. To the extent possible differences in characteristics were controlled for in the quantitative analysis, however.

By the time the quantitative and qualitative data were collected, performance payments had stopped for one month and 8 months respectively. According to interviews, many of the original workers had subsequently left following the removal of PBF. Stronger reactions may therefore have been observed had interviews occurred immediately after the withdrawal, however, of concern is that discontent persisted amongst the previous PBF group long afterwards. Furthermore, the study was unable to disentangle the influence of removing the fixed payment from the performance-based component. This was investigated previously by Huillery and Seban, where they found the removal of a performance-related payment compared with a fixed payment of the same amount had a more profound negative impact on motivation.^16^

The study was subject to other biases, including social desirability bias where respondents’ perceptions of what constitutes an acceptable answer or what they think the researcher wishes to hear may have influenced their responses. The analysis could have been strengthened had we been able to link the motivation scores to performance, however data on the latter was not captured. Due to resource constraints, qualitative interviews were only conducted in one province so may not be generalisable to the other sampled provinces. Kasai Occidental province was chosen because it would be easiest to access both workers who had either previously been exposed to the PBF model or never received PBF. However, a limitation is that the number of qualitative interviews undertaken overall was small. Although there are no established guidelines on sample sizes for qualitative interviews, the literature suggests between 20 and 50 interviews are often needed.^78,79^ Wewere limited in the number of interviews possible by the realities of a very difficult context (security, accessibility, availability of staff).

Finally, our confidence in comparing scores for dimensions exhibiting strong measurement invariance across PBF groups was higher compared to those constructs which showed weak or no invariance; the small sample of workers in the previous PBF groups would have affected the precision of measurement invariance testing.^33^

Controlled for health worker and health facility characteristics. Total number of observations for each regression was 392 due to missing values for certain characteristics.

## Conclusion

Programmes unable to sustain donor-funded payments to health workers should consider the consequences withdrawal could have for health worker motivation, and institute measures to mitigate against any adverse effects. Governments and donors designing new PBF schemes should develop realistic exit strategies if they are unlikely to be able to sustain these schemes over the longer-term.

## Acknowledgements

We would like to acknowledge the contribution of Dr Natasha Palmer from the London School of Hygiene and Tropical Medicine, London, UK to the design of the study. We also thank the Kinshasa School of Public Health, Kinshasa, Democratic Republic of the Congo and Ann-Marie Yongho and Paul-Samson Lusamba-Dikassa of Tulane International (TILLC) who oversaw the implementation of the health facility and health workers surveys, and Cele Manianga who assisted the collection of data for the qualitative component. Finally, we would like to thank Gavin Cochrane for his help in producing the figures.

## Ethical issues

The study received ethical approval from the Tulane University Institutional Review Board (Reference number: 14-633280), the Kinshasa School of Public Health Ethics Committee (Reference number: ESP/CE/024/2014), and the London School of Hygiene and Tropical Medicine Research Ethics Committee (Reference number: 8475). Informed written consent was obtained from all participating healthcare providers.

## Competing interests

The lead author has recently been appointed as a health adviser for DFID. DFID funded the health systems strengthening programme described in the article. No other competing interests have been declared.

## Authors’ contributions

RM conceived the study, and designed it in collaboration with JB and DRH. JL assisted RM in the analysis and interpretation of the quantitative data. RM also designed, collected, and analysed the qualitative data of the study. SMJ and JB helped with the interpretation of the qualitative data. RM drafted the initial manuscript, and all authors helped to revise it critically for intellectual content. All authors read and approved the final the manuscript.

## Funding

This work was supported by the UK Government’s Department for International Development.

## Authors’ affiliations

^1^Faculty of Public Health Policy, London School of Hygiene and Tropical Medicine, London, UK. ^2^Faculty of Medicine, Heidelberg Institute of Global Health, Heidelberg University, Heidelberg, Germany. ^3^School of Public Health and Tropical Medicine, Tulane University, New Orleans, LA, USA.

## Supplementary files

Supplementary files 1, 2, 3, and 4 contain Tables S1, S2, S3 and S4, respectively.

## Key messages

**Implications for policy makers**

- This research indicates that the withdrawal of donor-funded payments may have harmful repercussions for health worker motivation and service delivery.
- Programmes unable to sustain donor-funded payments to health workers should develop realistic exit strategies and institute measures to mitigate against any adverse effects on motivation prior to withdrawal.
- Governments and donors designing new performance-based financing (PBF) schemes or programmes involving supplemental payments to workers should consider whether the short-term advantages of introducing additional health worker payments outweigh the potential adverse long-term consequences in the event that donor financing ceases.

**Implications for public**

This research has demonstrated the potential negative implications for health worker motivation when donor-funded payments provided through a performance-based financing (PBF) scheme are withdrawn. PBF is increasingly being employed by the international community as a mechanism to enhance worker motivation and performance in fragile settings. However, the long-term funding for PBF is not always guaranteed. This study warrants consideration by those either designing or considering how to exit from a PBF scheme or programme administering supplemental payments to workers.

## Footnotes

[1] In July 2015, the provinces of the DRC underwent ‘decoupage,’ whereby the DRC’s 11 provinces were further subdivided into 26. This paper refers to the provinces prior to decoupage.
