# Supplementary material for: What Happens When Donors Pull Out? Examining Differences in Motivation Between Health Workers Who Recently Had Performance-Based Financing (PBF) Withdrawn With Workers Who Never Received PBF in the Democratic Republic of Congo
Source: Int J Health Policy Manag. 2019 Jul 13;8(11):646–61. doi: 10.15171/ijhpm.2019.55 (PMC6885854; doi:10.15171/ijhpm.2019.55)
Supplement: Supplementary file 1 [file ijhpm-8-646-s001.pdf]

Supplementary file 1

Table S1. Dimensions and distribution of item scores overall and by PBF status

		**Overall**	**Non PBF**	**Previous PBF**	**Total number obs**
**Dimension**	**Item**	**Mean, SD (median)**	**Mean, SD (median)**	**Mean, SD (median)**	
Conscientious ness	*I am confident about my ability to handle my work*	4.21, 0.46 (4)	4.23, 0.50 (4)	4.14, 0.35 (4)	452
	I effectively cope with any new challenges that occur in my work life	4.06, 0.50 (4)	4.09, (0.50) (4)	3.99, 0.50 (4)	452
	At work you can always depend on me	4.13, 0.46 (4)	4.13, 0.46 (4)	4.06, 0.46 (4)	452
	My work is always of high quality	4.02, 0.58 (4)	4.05, 0.57 (4)	3.93, 0.60 (4)	452
	I am a hard worker	4.15, 0.60 (4)	4.16, 0.56 (4)	4.10, 0.70 (4)	452
	I always arrive on time to work	4.05, 0.66 (4)	4.07, 0.67 (4)	3.99, 0.63 (4)	452
	I spend my time at work on work-related activities	4.13, 0.41 (4)	4.15, 0.39 (4)	4.08, 0.46 (4)	452
	I am careful not to make mistakes at work	4.18, 0.44 (4)	4.21, 0.47 (4)	4.12, 0.32 (4)	452
	When I am not sure how to treat a patient's condition I look for information or ask for advice	4.12, 0.50 (4)	4.14, 0.52 (4)	4.08, 0.42 (4)	452
	I do things which need to be done without being asked or told	4.06, 0.57 (4)	4.09, 0.53 (4)	3.97, 0.67 (4)	452
Pride	*This facility has a good reputation in the community*	4.12, 0.69 (4)	4.18, 0.67 (4)	3.95, 0.71 (4)	451
	It is a source of pride to work here	3.92, 0.80 (4)	3.96, 0.79 (4)	3.80, 0.69 (4)	451
	I feel I am doing something important in this work	3.95, 0.72 (4)	4.00, 0.70 (4)	3.80, 0.76 (4)	451
	The other workers are proud to deliver good care to patients	4.08, 0.62 (4)	4.13, 0.62 (4)	3.95, 0.61 (4)	451
Training	*How do you rate your opportunities to upgrade your skills and knowledge?*	3.39, 1.10 (4)	3.51, 1.06 (4)	3.06, 1.13 (3)	452
	How do you rate your ability to put into practice what you have learned from training?	3.84, 0.86 (4)	3.95, 0.75 (4)	3.54, 1.06 (4)	452
	How do you rate how you and your colleagues are chosen to attend training?	3.29, 1.05 (4)	3.38, 1.02 (4)	3.03, 1.09 (3)	452
	I have received sufficient training to do my job well	3.79, 0.96 (4)	3.83, 0.95 (4)	3.68, 1.00 (4)	451
Tasks	*How do you rate the description of your job role and tasks?*	3.76, 0.85 (4)	3.75, 0.88 (4)	3.80, 0.75 (4)	453
	How do you rate satisfaction with your workload?	3.29, 1.03 (4)	3.19, 1.06 (4)	3.60, 0.87 (4)	453
	How do you rate the division of work between you and your colleagues?	3.71, 0.79 (4)	3.68, 0.81 (4)	3.79, 0.71 (4)	443
	How do you rate the division of work between caring for patients and other tasks?	3.67, 0.81 (4)	3.59, 0.87 (4)	3.86, 0.57 (4)	453
	How do you rate the variety of your tasks?	3.67, 0.81 (4)	3.64, 0.85 (4)	3.75, 0.67 (4)	453
Availability of equipment /supplies	*How do you rate the availability of medicines in the health facility?*	2.34, 1.17 (2)	2.59, 1.17 (2)	1.60, 0.81 (1)	453

	How do you rate the availability of equipment in the health facility?	2.07, 1.03 (2)	2.16, 1.02 (2)	1.81, 1.03 (1.5)	452
	How do you rate the availability of medical supplies in the health facility?	2.44, 1.16 (2)	2.64, 1.18 (2)	1.88, 0.90 (2)	453
Income reflects effort	*The effort that we at this facility put into this job is reflected in our pay*	2.20, 1.02 (2)	2.37, 1.08 (2)	1.74, 0.59 (2)	451
	My job offers adequate pay compared with similar jobs	2.09, 0.93 (2)	2.24, 0.99 (2)	1.64, 0.55 (2)	451
	The income I receive is a fair reflection of my skills, knowledge and training	1.80, 0.71 (2)	1.89, 0.73 (2)	1.54, 0.56 (2)	448
Sufficiency of income	*The income that I receive from working at this facility more than covers my basic needs such as food, transport, and accommodation*	1.71, 0.65 (2)	1.79, 0.67 (2)	1.47, 0.55 (1)	448
Organization-al culture	*How do you rate your working relationships with upper-level staff?*	3.93, 0.73 (4)	3.96, 0.75 (4)	3.84, 0.68 (4)	453
	How do you rate your working relationship with colleagues?	4.09, 0.66 (4)	4.15, 0.63 (4)	3.92, 0.71 (4)	453
	How do you rate the transparency of the management of financial resources by the facility?	3.49, 1.04 (4)	3.49, 1.07 (4)	3.48, 0.98 (4)	452
	How do you rate your participation in decision-making to resolve problems in the facility?	3.80, 0.76 (4)	3.85, 0.76 (4)	3.65, 0.73 (4)	453
Community relationships	*How do your rate your relationship with local leaders in the community*	4.00, 0.72 (4)	4.04, 0.74 (4)	3.91, 0.64 (4)	451
Turnover intention	*I intend to leave this facility as soon as I can find another position*	3.00, 1.25 (4)	3.07, 1.21 (4)	2.82, 1.34 (3)	449
Extrinsic motivation	*I only do this job so that I get paid at the end of the month*	3.62, 1.07 (4)	3.50, 1.07 (4)	3.95, 0.99 (4)	451
