# Supplementary material for: What Happens When Donors Pull Out? Examining Differences in Motivation Between Health Workers Who Recently Had Performance-Based Financing (PBF) Withdrawn With Workers Who Never Received PBF in the Democratic Republic of Congo
Source: Int J Health Policy Manag. 2019 Jul 13;8(11):646–61. doi: 10.15171/ijhpm.2019.55 (PMC6885854; doi:10.15171/ijhpm.2019.55)
Supplement: Supplementary file 2 [file ijhpm-8-646-s002.pdf]

Supplementary file 2

Table S2. Original survey items and explanation around why certain items were dropped from the confirmatory factor analysis

Dimension	Question	Item included (Y/N)	Reason why item was dropped
Income reflects effort	The effort that we at this facility put into this job is reflected in our pay	Y	
	My job offers adequate pay compared with similar jobs	Y	
	The income I receive is a fair reflection of my skills, knowledge and training	Y	
	How do you rate your salary with respect to your workload?	N	High number of not applicable answers received as salaries not given to all staff. This was identified post distribution of the survey.
Sufficiency of income	The income that I receive from working at this facility more than covers my basic needs such as food, transport, and accommodation	Y	
	With this job I have worries about how to support myself and my family	N	Did not group well with sufficiency of income dimension when performing psychometric item analysis.
	How do you rate your salary with respect to your competencies/ability?	N	High number of not applicable answers received as salaries not given to all staff. This was identified post distribution of the survey.
	How do you rate your salary with respect to your allowances (travel allowance, bonus, medical care)?	N	High number of not applicable answers received as salaries not given to all staff. This was identified post distribution of the survey.
	How satisfied are you with the system of compensation/motivation of personnel?	N	Question was deemed to be too vague and did not fit theoretically with the proposed dimension.
Availability of equipment/supplies	How do you rate the availability of medicines in the facility?	Y	
	How do you rate the availability of equipment in the facility?	Y	
	How do you rate the availability of medical supplies in the facility?	Y	
	How do you rate the physical condition of the facility building?	N	This question was to be grouped with questions above under a wider dimension of "resources". However, responses were very different compared to other items under resources (now availability of equipment/medical supplies) so was not included.
	How do you rate the number of personnel working in the facility?	N	This question was to be grouped with questions above under a wider dimension of "resources". However, responses were very different compared to other items under

			resources (now availability of equipment/medical supplies) so was not included.
Training	How do you rate your ability to put into practice what have you learned from training?	Y	
	How do you rate how you and your colleagues are chosen to attend training?	Y	
	How do you rate your opportunities to upgrade your skills and knowledge?	Y	
	I have received sufficient training to be able to perform my job well	Y	
Tasks	How do you rate your satisfaction with your workload?	Y	
	How do you rate the division of work between you and your colleagues?	Y	
	How do you rate the division of work between caring for patients and other tasks?	Y	
	How do you rate the variety of your tasks?	Y	
	How do you rate the description of your responsibilities and your tasks?	Y	
	How do you rate the flexibility with attendance and work hours?	N	The tasks dimension was initially wider to include questions on workload. However, this item did not correspond well with other items under the "tasks" dimension when measuring the mean distribution of responses and cronbach's alpha.
	How do you rate the help you receive from other members of your team?	N	The tasks dimension was initially wider to include questions on workload. However, this item did not correspond well with other items under the "tasks" dimension when measuring the mean distribution of responses and cronbach's alpha.
	How do you rate your level of responsibility?	N	Did not correspond well with other items under "tasks" dimension when measuring mean distribution of responses and cronbach alpha.
	How do you rate the stability of your contract?	N	Dropped due to high level of not applicable responses, as formal contracts did not tend to operate in health facilities. Also, did not fit well conceptually with the dimension.
Pride	This facility has a good reputation in the community	Y	
	It is a source of pride to get a job at this facility	Y	
	In this facility, providers are proud to deliver good services to patients	Y	

	I feel that I am doing something important in this job	Y	
Community relationships	How do you rate your relationship with local leaders of the community?	Y	
	How do you rate your ability to satisfy the needs of the community?	N	Initially belonged to a differently defined dimension called 'community factors' – but items were too disparate and did not group well in the analysis – so decision to change the dimension to community relationships.
	How do you rate your safety and security to live and practice in the community	N	Initially belonged to a differently defined dimension called 'community factors' – but items were too disparate and did not group well in the analysis – so decision to change the dimension to community relationships
	How do you rate your respect from the community?	N	Initially belonged to a differently defined dimension called 'community factors' – but items were too disparate and did not group well in the analysis – so decision to change the dimension to community relationships
Organisational culture	How do you rate your working relationships with upper-level staff?	Y	
	How do you rate your professional relationships with your colleagues?	Y	
	How do you rate the transparency of the management of financial resources by the facility?	Y	
	How do you rate your involvement in decisions to resolve problems within the facility?	Y	
	How do you rate the level of respect accorded to you by your internal supervisors in the facility?	N	High number of not applicable answers received, as sometimes the most senior member of staff was interviewed at the facility.
	How do you rate the management of the facility by the MSP or health zone office?	N	High number of not applicable answers received, as not all staff interacted with health office officials.
Conscientiousness	I spend my time at work on work-related activities	Y	
	I do things which need to be done without being asked or told	Y	
	When I am not sure how to treat a patient's condition I look for information or ask for advice	Y	
	I am careful not to make errors at work	Y	
	I am a hard worker	Y	
	My work is consistently of a high quality	Y	

	At work you can always depend on me	Y	
	I effectively cope with any new challenges that occur in my work life	Y	
	I am confident about my ability to handle my work	Y	
	I always arrive on time to work	Y	
	I am rarely absent from work	N	Did not correspond well with other items under “conscientiousness” dimension when measuring mean distribution of responses and cronbach alpha.
Extrinsic motivation	I only do this job so that I get paid at the end of the month	Y	
Turnover intention	I intend to leave this facility as soon as I can find another position	Y	
Respect /recognition	How do you rate the level of respect accorded to you by your external supervisors in the facility?	N	Partially captured under organisational culture, and did not form a stable construct on its own.
	How do you rate the recognition by your superiors for a job well done?	N	Partially captured under organisational culture, and did not form a stable construct on its own.
Self-efficacy	I feel that I have control of things concerning my work	N	Initially included under a different dimension of self-efficacy. However, this dimension was dropped as cronbach alpha was poor when grouped with other items presumed to be measuring self-efficacy (indicating very different scoring so potentially not measuring the same construct).
	I feel that at work things are going the way I would like them to	N	Initially included under a different dimension of self-efficacy. However, this dimension was dropped as cronbach alpha was poor when grouped with other items presumed to be measuring self-efficacy (indicating very different scoring so potentially not measuring the same construct).
No pre-defined dimension (added by development partners)	I would recommend this profession to my children	N	This item was suggested by development partners but did not correspond with any pre-defined dimension.
	How do you rate your opportunities for promotion?	N	This item was suggested by development partners but did not correspond with any pre-defined dimension.

In summary, 60 items were initially included in the questionnaire. Two of the items which were added by development partners, were not hypothesised to be associated with a dimension a priori, so were not included in the analysis. This left 58 items for confirmatory factor analysis.

However, on initial analysis of the data, it was found that six of the items had a high level of not-applicable responses (greater than 10%). On further review, it was identified that they may not have been applicable to all workers e.g. not all workers received a government salary, so these items were dropped.

For the rest of the items, psychometric item analysis examining item distributions, summary statistics and correlation patterns was undertaken. This involved checking mean distribution and standard deviation of item scores, as well as cronbach’s alpha. Ten items did not correspond well with other items pertaining to their intended dimension and so were dropped from the analysis. A further two dimensions, namely ‘self-efficacy’ and ‘respect/recognition’ were dropped as well as they did not perform well as constructs in the confirmatory factor analysis.
