# Supplementary material for: What Happens When Donors Pull Out? Examining Differences in Motivation Between Health Workers Who Recently Had Performance-Based Financing (PBF) Withdrawn With Workers Who Never Received PBF in the Democratic Republic of Congo
Source: Int J Health Policy Manag. 2019 Jul 13;8(11):646–61. doi: 10.15171/ijhpm.2019.55 (PMC6885854; doi:10.15171/ijhpm.2019.55)
Supplement: Supplementary file 3 [file ijhpm-8-646-s003.pdf]

Supplementary file 3

Table S3. Hypothesised relationship of different demographic and health facility characteristics with health worker motivation

Variables	Hypothesised relationship with motivation
**Age**	In a study by Prytherch et al.,[1] older respondents were significantly more positive in their responses to questions assessing their level of motivation. The authors postulated that older workers may receive greater respect and appreciation for their work from the community and colleagues given their greater level of experience. In other studies, older workers have been found to be more committed to working in the facility than younger workers, and more satisfied with their work overall.[2,3]
**Sex**	A study in Zambia found female public sector health workers to be less satisfied than male workers.[4] However, another study found no difference in job satisfaction between males and females when controlling for other socio-demographic variables.[2]
**Number of dependents**	In Tanzania, it was found that the more dependents a health worker had, the more positive they were in response to questions concerning their motivation.[1] This may be because the income of workers becomes more important as their number of dependents increases making workers less likely to respond negatively to these questions. Workers with children have also been found to be significantly more committed to staying in a facility compared to workers without children.[3]
**Urban-rural status**	Opportunities, for example for career development, may be greater in urban areas compared to rural areas which could have an effect on motivation.[5] Developing countries often experience 'urban bias' whereby urban areas experience a greater provision of services and investment compared to rural areas.[6] The relative underinvestment in rural areas may serve to reduce the motivation of workers.
**Total number of staff delivering healthcare present on the day**	The number of staff working in a facility at a given time will affect the distribution of tasks and potentially the workload of personnel. This may in turn affect health worker motivation. Workload is an important motivating factor, particularly in the context of limited resources.[7]
**Number of services offered**	The more services offered by a facility, the greater the potential for workers to use a variety of skills and have responsibility for certain tasks. This may have the potential to affect motivation, according to the job characteristics model developed by Hackman and Oldham.[8]
**Distance of the facility from the village**	Distance of the facility from the village has been used here as a proxy for the remoteness of facilities. Globally, it is challenging to recruit and retain workers in remote areas .[9] Nonetheless, Stilwell found that health workers based in remote areas of Zimbabwe displayed a high level of motivation despite a lack of financial incentives compared to workers in less remote areas .[10]
**Education**	Educational background has been shown to be a predictor of intention to leave a health facility.[11]
**Years in position**	In Tanzania, respondents who had been working for longer tended to be less critical about the management of the facility. This may be due to their understanding and acceptance over time around the constraints faced by facilities.[1] However, another study in Afghanistan showed that workers who had been working longer at a facility, had a lower intent to stay than those who had been working for a shorter period of time.[3]
**Type of facility**	A cross-country analysis of Tanzania, South Africa and Malawi indicated that workers in public hospitals were less satisfied compared to workers in clinics or health centres.[2] Reference facilities in the DRC are bigger than health centres and offer a broader range of services which may affect the motivation of workers.
**Total population of the village**	The greater the population served by a facility may result in more patients accessing the facility. The increased workload may in turn affect staff motivation.

References

1. Prytherch H, Kagone M, Aninanya GA, et al. Motivation and incentives of rural maternal and neonatal health care providers: a comparison of qualitative findings from Burkina Faso, Ghana and Tanzania. *BMC Health Serv Res.* 2013;13:149. doi:10.1186/1472-6963-13-149

2. Blaauw D, Ditlopo P, Maseko F, et al. Comparing the job satisfaction and intention to leave of different categories of health workers in Tanzania, Malawi, and South Africa. *Glob Health Action.* 2013;6:19287.

3. Fogarty L, Kim YM, Juon H-S, et al. Job satisfaction and retention of health-care providers in Afghanistan and Malawi. *Hum Resour Health.* 2014;12(1):11.

4. Gow J, George G, Mwamba S, Ingombe L, Mutinta G. Health worker satisfaction and motivation: an empirical study of incomes, allowances and working conditions in Zambia. *International Journal of Business and Management.* 2012;7(10):37.

5. Kotzee T, Couper I. What interventions do South African qualified doctors think will retain them in rural hospitals of the Limpopo province of South Africa. *Rural Remote Health.* 2006;6(3):581.

6. Lipton M. Why poor people stay poor: a study of urban bias in world development: London: Canberra: Temple Smith; Australian National University Press; 1977.

7. World Health Organization. The world health report: working together for health 2006. http://www.who.int/whr/2006/en/. Accessed 10 November 2017.

8. Hackman JR, Oldham GR. Motivation through the design of work: Test of a theory. *Organ Behav Hum Perform.* 1976;16(2):250-279.

9. Rourke J. WHO Recommendations to improve retention of rural and remote health workers-important for all countries. *Rural Remote Health.* 2010;10(4):1654.

10. Stilwell B. Health worker motivation in Zimbabwe. 2001. *World Health Organization, Geneva Google Scholar.*

11. Tzeng H-M. The influence of nurses' working motivation and job satisfaction on intention to quit: an empirical investigation in Taiwan. *International journal of nursing studies.* 2002;39(8):867-878.
