# Supplementary material for: What Happens When Donors Pull Out? Examining Differences in Motivation Between Health Workers Who Recently Had Performance-Based Financing (PBF) Withdrawn With Workers Who Never Received PBF in the Democratic Republic of Congo
Source: Int J Health Policy Manag. 2019 Jul 13;8(11):646–61. doi: 10.15171/ijhpm.2019.55 (PMC6885854; doi:10.15171/ijhpm.2019.55)
Supplement: Supplementary file 4 — Supplementary files 1, 2, 3, and 4 contain Tables S1, S2, S3 and S4, respectively. [file ijhpm-8-646-s004.pdf]

Supplementary file 4

Table S4. Proportion of nurses receiving sources of income and mean and median values of income received by PBF status

	**Workers not receiving PBF (n=302)**			**Workers receiving PBF (n=105)**		
**Source of income**	**Overall proportion of workers who received source of income**	**Median income per month among those receiving income in USD (IQR)**	**Mean income per month among those receiving income in USD (Standard Error)**	**Overall proportion of workers who received source of income**	**Median income per month among those receiving income in USD (IQR)**	**Mean income per month among those receiving income in USD (Standard Error)**
**Payments from government**						
Salary from government	33.4%	42.25 (22-75)	53.43 (49.78)	24.8%	70.16 (47-85)	76.06 (89.92)
Occupational risk allowance from government	50.0%	12.89 (10-30)	46.60 (86.32)	61.0%	11.92 (11-13)	14.43 (11.79)
**Payments from other sources**						
User fees	69.1%	28.71 (16-68)	96.93 (185.10)	90.5%	10.83 (8-16)	14.85 (15.69)

Gifts/informal payments from patients	15.6%	3.25 (2-11)	9.26 (11.66)	20.0%	5.42 (3-8)	7.58 (7.25)
Per diems	55.2%	4.06 (2-8)	9.30 (29.47)	41.9%	4.11 (1-6)	5.78 (6.19)
Income from private clinical practice	5.6%	17.61 (5-22)	28.13 (33.69)	11.4%	21.67 (18-62)	40.90 (34.61)
Income from supplemental (non-clinical) activities	38.9%	86.67 (54-173)	142.75 (151.36)	69.2%	43.34 (27-87)	91.46 (154.97)
**Total income**	N/A	84.57 (33-199)	178.96 (244.53)	N/A	84.67 (40-135)	118.63 (167.35)

N.B. For the occupational risk allowance, one outlier income was dropped from the analysis; no outliers were detected for any other income amount.

The table above illustrates differences in the level of income received by nurses who previously PBF compared to those who did not. The proportion of nurses receiving salaries was lower for those who received donor-funding in the past, but median and mean amounts were higher. Nurses who had previously received PBF were more likely to receive income from user fees, albeit a smaller median and mean amount compared with those who had not been receiving donor payments. They were also more likely to engage in private clinical practice and other non-clinical activities to supplement their income. Despite the differences, however, both median and mean overall total income were very similar for both groups. The minimum daily wage in the DRC is $1.83, or $298 per year.[1]

[1] US Department of State. Bureaucracy of Democracy, Human Rights, and Labor. "Country Reports on Human Rights Practices for 2017. *Available at:* https://www.state.gov/j/drl/rls/hrrpt/humanrightsreport/index.htm#wrapper. *Updated 19 January 2019. Accessed: 19 January 2019.*
